# Treating Sleep Problems in Young People at Ultra-High Risk of Psychosis: A Feasibility Case Series

**DOI:** 10.1017/S1352465817000601

**Published:** 2017-10-30

**Authors:** Jonathan Bradley, Daniel Freeman, Eleanor Chadwick, Allison G. Harvey, Bradley Mullins, Louise Johns, Bryony Sheaves, Belinda Lennox, Matthew Broome, Felicity Waite

**Affiliations:** Department of Psychiatry, University of Oxford; Sleep and Circadian Neuroscience Institute, University of Oxford; and Oxford Health NHS Foundation Trust; Department of Psychiatry, University of Oxford; Sleep and Circadian Neuroscience Institute, University of Oxford; and Oxford Health NHS Foundation Trust; Department of Psychiatry, University of Oxford; Department of Psychology, UC Berkeley; Oxford Health NHS Foundation Trust; Oxford Health NHS Foundation Trust; Department of Psychiatry, University of Oxford; Sleep and Circadian Neuroscience Institute, University of Oxford; and Oxford Health NHS Foundation Trust; Oxford Health NHS Foundation Trust; Department of Psychiatry, University of Oxford; Oxford Health NHS Foundation Trust; and Faculty of Philosophy, University of Oxford; Department of Psychiatry, University of Oxford; Sleep and Circadian Neuroscience Institute, University of Oxford; and Oxford Health NHS Foundation Trust

**Keywords:** sleep, insomnia, psychosis, ultra-high risk of psychosis, at-risk mental state

## Abstract

**Background:** Our view is that sleep disturbance may be a contributory causal factor in the development and maintenance of psychotic experiences. A recent series of randomized controlled intervention studies has shown that cognitive-behavioural approaches can improve sleep in people with psychotic experiences. However, the effects of psychological intervention for improving sleep have not been evaluated in young people at ultra-high risk of psychosis. Improving sleep might prevent later transition to a mental health disorder. **Aims:** To assess the feasibility and acceptability of an intervention targeting sleep disturbance in young people at ultra-high risk of psychosis. **Method:** Patients were sought from NHS mental health services. Twelve young people at ultra-high risk of psychosis with sleep problems were offered an eight-session adapted CBT intervention for sleep problems. The core treatment techniques were stimulus control, circadian realignment, and regulating day-time activity. Participants were assessed before and after treatment and at a one month follow-up. **Results:** All eligible patients referred to the study agreed to take part. Eleven patients completed the intervention, and one patient withdrew after two sessions. Of those who completed treatment, the attendance rate was 89% and an average of 7.6 sessions (*SD* = 0.5) were attended. There were large effect size improvements in sleep. Post-treatment, six patients fell below the recommended cut-off for clinical insomnia. There were also improvements in negative affect and psychotic experiences. **Conclusion:** This uncontrolled feasibility study indicates that treating sleep problems in young people at ultra-high of psychosis is feasible, acceptable, and may be associated with clinical benefits.

## Introduction

There is increasing recognition that sleep disturbance is a contributory causal factor for many mental health problems (Harvey, 2009). Our group have conducted a programme of research specifically on sleep and psychotic experiences (Freeman et al., 2009). This has tested the potential causal role of sleep in psychosis (e.g. Freeman et al., 2012b; Reeve et al., 2015, 2017; Sheaves et al., 2016) and, in two randomized controlled trials, the effects of psychological sleep interventions for individuals with psychotic experiences (Freeman et al., 2015, 2017). Psychotic experiences exist on continua of severity and distress (van Os et al., 2009). We have demonstrated the benefit of cognitive behavioural therapy (CBT), the first-line treatment of choice for improving sleep (NICE, 2015), across a number of points of the continuum of psychotic experiences. We have shown large effect size improvements in sleep in patients with persistent delusions and hallucinations (Freeman et al., 2015). In a randomized controlled trial with almost four thousand students with insomnia, we have definitively shown that treating sleep leads to a reduction in (non-clinical) paranoia and hallucinations (Freeman et al., 2017). The current study continues this course of research by investigating the feasibility of CBT for sleep problems in young people at ultra-high risk of psychosis. Treating sleep disturbance in this population could potentially contribute to preventing transition to psychosis.

The term ‘ultra-high risk of psychosis’ refers to a cluster of subthreshold psychotic-like experiences associated with a high risk of onset of psychotic disorder in the near future (Fusar-Poli et al., 2012; Yung et al., 2003, 2005). Psychotic-like experiences differ from psychotic symptoms in their intensity, frequency or duration. The persistence of these psychotic-like experiences constitutes a risk factor for the development of clinical psychosis (Dominguez et al., 2011). Thus early intervention and targeted prevention are warranted.

Sleep disturbance predicts the occurrence and persistence of psychotic-like experiences in adolescents (Lee et al., 2012) and young people who are assessed as at ultra-high risk of psychosis (Lunsford-Avery et al., 2015) as well as specific psychotic experiences, including paranoia (Freeman et al., 2009, 2012a) and hallucinations (Reeve et al., 2015; Sheaves et al., 2016). In addition, sleep disturbance has been identified as a predictor of transition to psychosis (Ruhrmann et al., 2010) and students who received treatment for insomnia were less likely to meet criteria for ultra-high risk of psychosis (Freeman et al., 2017). Thus the treatment of sleep disturbance might have secondary benefits for wider mental health in young people at ultra-high risk of psychosis.

Adolescence is a key period for intervention. Firstly, adolescence is the peak age of onset for mental health problems, including psychosis (Kessler and Wang, 2008). Secondly, there may be a particular risk for the onset of sleep problems due to a combination of biological, social and environmental changes which occur during this period (Carskadon, 2011; Harvey, 2016). A shift in circadian rhythms following puberty interacts with social and behavioural changes in adolescence (such as reduced parental oversight and increased use of social media) to delay the sleep phase. The inability to delay the sleep-offset time due to school or work, for example, results in a reduced sleep window and can precipitate sleep problems. Thus sleep dysfunction is a widespread problem in adolescence (Johnson et al., 2006; Ohayon, 2002) associated with a number of mental health problems (Morrison et al., 1992) and a wide-ranging impact on wellbeing, from work and social function (Gradisar et al., 2011) to suicidal thoughts and self-harm behaviour (Wong et al., 2011).

CBT has been established as the first-line treatment of choice for improving sleep and has a strong evidence base in adults (Mitchell et al., 2012). Despite this, the evidence base in young people is sparse. A handful of studies have indicated that, with adaptions, CBT for insomnia may be a fruitful approach to improving sleep problems in adolescents (Bootzin and Stevens, 2005; de Bruin et al., 2015; Schlarb et al., 2010). However, the evidence base in the adolescent phase of lifespan (14–24 years) remains underdeveloped and no study has yet sought to treat sleep problems in adolescents at ultra-high risk of psychosis.

The current study builds on our previous work treating sleep problems in people with psychosis and subthreshold psychotic experiences. In this study the treatment is tailored towards young people as this encompasses the peak age of onset for mental health problems. The primary aim was to assess the feasibility and acceptability of our adapted sleep intervention through recruitment rate, treatment uptake, and identification of the modules used. The secondary aim was to gain preliminary outcome data for sleep, psychotic experiences, and psychological wellbeing.

## Method

### Participants

Twelve participants were recruited from child and adolescent mental health services or early intervention in psychosis services in Oxford Health NHS Foundation Trust. The inclusion criteria were: current sleep problems (defined as a score >15 on the Insomnia Severity Index *or* on the SLEEP-50, a score >15 on the impact subscale *and* score >19 on the insomnia subscale *or* score >8 on the circadian rhythm sleep disorder scale); meeting criteria for ultra-high risk of psychosis based on attenuated psychosis (see Yung et al., 2006 for full criteria) on the Comprehensive Assessment of At-Risk Mental States (CAARMS); help seeking (i.e. had contacted mental health services); and aged 14–24 years. The exclusion criteria were: any diagnosis of psychosis; a primary diagnosis of alcohol or substance dependency, personality disorder, organic syndrome or learning disability; sleep apnoea; insufficient command of English for engaging in therapy; and current engagement in any other individual psychological therapy. One participant completed the baseline but discontinued therapy after two sessions without giving a reason. This participant did not complete any follow-up assessments and therefore the data are not presented in the results section.

### Design

The study had an A–B design. Quantitative assessments were completed at baseline, post-therapy, and at one month follow-up. A qualitative interview was completed around one week after the follow-up (data from the qualitative interviews to be published separately). Treatment lasted up to 10 weeks for up to eight sessions. Participation for the study typically lasted four months.

### Assessments

All measures were used at all assessment points. All assessments were conducted by a research assistant (E.C., B.M.) with the exception of four baseline assessments which were conducted by a clinical psychologist (J.B.). Sleep disturbance was measured using three scales. The SLEEP-50 (Spoormaker et al., 2005) assesses DSM-IV sleep disorders across nine subscales. At post-therapy and follow-up, only the Insomnia, Circadian Rhythm Disruption, Factors Influencing Sleep and Impact subscales were used as these were the main targets for therapy. The Insomnia Severity Index (ISI; Bastien, 2001) specifically assesses insomnia and has a recommended cut-off score of nine or above for detection of insomnia in adolescents (Chung et al., 2011) and fifteen or above for detection of insomnia in adults. For the measurement of sleep quality, the Pittsburgh Sleep Quality Index (Buysse et al., 1989) was used. Sleep diaries and actigraphy were also used for one week following each assessment to capture subjective and (potentially) objective measurements of daily sleep patterns.

Attenuated psychotic experiences were measured using the Green Paranoid Thoughts Scale (GPTS; Green et al., 2008) and the hallucinations subscale of the Specific Psychotic Experiences Questionnaire (Ronald et al., 2013). A structured interview using the CAARMS (Yung et al., 2005) was carried out to determine eligibility and whether participants transitioned to psychosis only. Negative affect was measured using the Depression, Anxiety and Stress Scale (Lovibond and Lovibond, 1995). The Warwick–Edinburgh Mental Well-being Scale (Tennant et al., 2007) and the Work and Social Adjustment Scale (Mundt et al., 2002) were used to assess quality of life. Consideration was given to the appropriateness of all measures for use in this age range.

### Adverse events

Serious adverse events were defined as any untoward occurrence that results in death, is life threatening, requires in-patient hospitalization or results in persistent or significant disability. Formal complaints regarding the therapy and transition to psychosis were also recorded. Transition to psychosis was determined through multiple sources including: scoring above psychosis threshold on the CAARMS at the research assessments; review of clinical notes; team feedback; use of anti-psychotic medication and the clinical observation of the study therapist.

### The intervention

The intervention was delivered by a clinical psychologist (J.B.) with regular supervision from F.W. The treatment package, called SleepWell, incorporates CBT techniques that address insomnia (for example, stimulus control, sleep hygiene, relaxation), and those which address circadian rhythm disruption (for example, sleep/wake cycle realignment, daily activity), as recommended in transdiagnostic approaches to sleep disturbance in adolescence (Harvey, 2016; Harvey and Buysse, 2017). The intervention draws on CBT techniques that have been widely established as effective for reducing sleep disturbance in adults (Espie, 2006; Harvey et al., 2007) and adapted for patients with psychosis (Freeman et al., 2015; Waite et al., 2016). Formal sleep restriction was not included as part of the intervention due to the link between sleep loss and psychotic experiences. However, the intervention contained elements of sleep restriction, for example if participants slept beyond their target rise time due to delayed sleep onset, they were encouraged to set an alarm to wake them at the same time each day. As an illustration, a participant who was waking at 11.00 a.m. because they were not falling asleep until the early morning but wanted to wake at 9.00 a.m. was encouraged to set their alarm progressively earlier to compress the sleep window.

The intervention was typically delivered in one-hour sessions, once a week, for up to eight sessions. The intervention is manualized in a modular format, with the targets for treatment identified in an individualized formulation of the sleep problems. Actigraphy data were used in the intervention to monitor changes in sleep patterns and identify foci for change. Additional contact between sessions, via text message, telephone or email, was provided in order to support the implementation of treatment strategies.

Adaptations were made due to the biological, social and environmental differences between adolescents and adults. Firstly, attention was given to the shift in circadian rhythms that occurs from puberty and interacts with social and behavioural changes to delay the sleep phase and reduce the sleep window (Harvey, 2016). Secondly, consideration was given to the practical challenges of completing stimulus control, only using bed for sleeping, when the bedroom was the participant's only private, independent space or needed to be used for multiple purposes, for example study or homework. This required negotiation with other family members and highlighted how other members of the household could contribute to the intervention. Therefore, participants were encouraged to recruit a ‘sleep team’ of family, friends or partners who could support them in implementing techniques at home. Finally, the use of electronic devices at night, in particular to communicate with social networks, was addressed and changes in use negotiated to maximize the chances of getting good sleep.

### Analysis

The primary focus of this study was feasibility and acceptability of the intervention, hence the key outcomes concerned provision of descriptive statistics for recruitment rates, treatment uptake, and data completion. For clinical outcomes, paired *t*-tests were conducted to provide change scores and confidence intervals for comparisons between baseline and post-therapy and between baseline and follow-up. Following recommendations that pilot studies should focus on confidence interval estimation (Lancaster et al., 2004), *p-*values are not reported. Effect size estimates (*d*) were calculated by dividing the change scores from the *t*-tests by the standard deviation of the baseline scores. All statistical analysis was conducted using SPSS version 24.0 (IBM Corporation, 2016).

## Results

### Basic demographic and clinical information

Six participants were female and five were male. The average age of the participants at baseline was 18.5 years (*SD* = 1.9). Most participants were white (*n* = 10). The participants were predominantly students, either at school or sixth-form (*n* = 4) or in higher education (*n* = 3); the remaining participants were either in full-time (*n* = 2) or part-time (*n* = 2) employment. Three participants reported taking medication at least once in the past month to help them sleep. One patient had been taking a regular prescription of melatonin for two months prior to commencing the study, which continued at the same dose during their participation in the study.

### Recruitment rate

Twenty-nine patients were referred to the study. Three of these individuals were uncontactable and seven declined to be screened (one reported no sleep problems, one reported no subthreshold psychotic experiences, and five gave no specific reason). Nineteen patients were screened by a clinical psychologist (J.B.). Five were excluded due to current or recent experiences of psychosis and two were excluded due to falling below threshold either for ultra-high risk of psychosis (*n* = 1) or sleep problems (*n* = 1). After screening, all 12 participants who were invited to take part in the study consented to participate.

### Completion rate of assessments

The 11 participants who completed therapy attended all assessments. The data completion rate was very high: there was only one questionnaire for which one participant did not provide analysable data. For the sleep diaries, which were completed over the course of a week, the completion rate was much lower: six participants provided data for baseline and post-therapy time points, which was insufficient for analysis. Actigraphy was used by all participants; however, without the sleep diary data to provide estimations for the analytic program these data could not be reliably analysed.

### Uptake of the intervention

The average number of sessions delivered per participant was 7.6 (*SD* = 0.5; range 7–8) and the average session length was 61 minutes. For the 84 sessions delivered, there were three non-attendances and seven cancellations, yielding an attendance rate of 89%. All participants received modules on assessment and formulation, sleep hygiene, stimulus control, regulating the sleep cycle, day-time activity and relapse prevention. Other modules received by participants were overcoming sleep-related worry (*n* = 6), increasing motivation (*n* = 4), coping with voices (*n* = 3), managing nightmares (*n* = 1) and enhancing relaxation (*n* = 1). Guided relaxation recordings were provided to four other participants as part of regulating the sleep cycle. The average number of modules received was 6.3 (*SD* = 0.9; range 5–8) out of a possible eight.

### Adverse events

No participants transitioned to psychosis (as measured by the CAARMS) at either post-therapy or follow-up. There were no hospital admissions, formal complaints regarding the therapy, or serious adverse events reported.

### Clinical outcomes

Tables 1 and 2 show the means and standard deviations for the outcome measures. At the post-therapy assessment, six out of 11 participants fell below the recommended cut-off for insomnia on the ISI and nine fell below the study inclusion cut-offs on the ISI and SLEEP-50. Table 3 displays the change scores and the confidence intervals for the sleep outcome measures during the study. After therapy, there were reductions in insomnia as assessed by the SLEEP-50 (*d* = 1.7) and ISI (*d* = 6.8). There was also improvements on the Pittsburgh Sleep Quality Index (PSQI; *d* = 2.9), and the SLEEP-50 circadian rhythm disruption (*d* = 1.2) and impact (*d* = 2.9) subscales. These improvements in sleep were maintained at follow-up. Table 4 shows the change scores and the confidence intervals for the secondary measures. At post-therapy, improvements were observed in depression (*d* = 0.5), stress (*d* = 0.8), anxiety (*d* = 0.2), wellbeing (*d* = 0.7), and occupational and social functioning (*d* = 0.7). There was also evidence of improvements in levels of paranoia (*d* = 0.6) and hallucinations (*d* = 0.3).

**Table 1.** Descriptive statistics for sleep-specific outcome measures at each assessment point

	Mean score (*SD*)											
Assessment				SLEEP-50		SLEEP-50		SLEEP-50 Factors		SLEEP-50		PSQI
point	*n*	ISI	*n*	Insomnia	*n*	CRSD	*n*	influencing sleep	*n*	Impact	*n*	total
Baseline	11	17.2 (1.2)	11	22.6 (4.5)	11	7.1 (1.2)	11	13.2 (2.4)	11	21.6 (2.9)	11	12.5 (2.9)
Post-therapy	11	9.1 (5.1)	11	14.9 (4.4)	11	4.9 (1.6)	11	10.9 (2.1)	11	14.6 (4.8)	11	6.5 (3.2)
Follow-up	11	9.1 (4.6)	11	14.2 (3.3)	11	4.6 (1.9)	11	10.6 (1.8)	11	14.2 (3.5)	11	7.8 (3.2)

ISI, Insomnia Severity Index; PSQI, Pittsburgh Sleep Quality Index.

**Table 2.** Descriptive statistics for psychotic experiences and wellbeing outcome measures at each assessment point

	Mean score (*SD*)														
Assessment						DASS		DASS		DASS					
point	*n*	GPTS	*n*	SPEQ	*n*	Depression	*n*	Anxiety	*n*	Stress	*n*	WEMWBS	*n*	WSAS	*n*
Baseline	11	71.6 (22.7)	11	13.8 (5.5)	11	12.0 (6.0)	11	8.2 (4.7)	11	12.2 (4.8)	11	35.4 (7.9)	11	24.2 (7.5)	11
Post-therapy	11	58.3 (24.1)	11	11.9 (5.8)	10	8.3 (6.5)	10	6.2 (3.8)	10	8.2 (6.1)	11	41.1 (6.2)	11	18.6 (9.5)	11
Follow-up	11	53.9 (21.3)	11	9.4 (6.6)	11	7.8 (7.8)	11	7.3 (5.9)	11	8.2 (5.7)	11	42.1 (8.1)	11	16.6 (10.4)	11

GPTS, Green Paranoid Thoughts Scale; SPEQ, Specific Psychotic Experiences Questionnaire; DASS, Depression Anxiety Stress Scale; WEMWBS, Warwick–Edinburgh Mental Well-Being Scale; WSAS, Work and Social Adjustment Scale.

**Table 3.** Change scores and 95% confidence intervals for sleep-specific outcome measures

	Change scores											
			SLEEP-50		SLEEP-50		SLEEP-50 Factors		SLEEP-50			
	ISI		Insomnia		CRSD		influencing sleep		Impact		PSQI	
Comparison	Change	CI	Change	CI	Change	CI	Change	CI	Change	CI	Change	CI
Baseline *vs*	–8.1	–11.4,	–7.7	–10.3,	–2.2	–3.1,	–2.3	–3.8,	–6.9	–9.7,	–5.9	–8.5,
post-therapy		–4.8		–5.2		–1.2		–0.7		–4.1		–3.3
Baseline *vs*	–8.1	–11.1,	–8.5	–11.1,	–2.5	–3.6,	–2.5	–3.8,	–7.4	–9.2,	–4.6	–7.0,
follow-up		–5.0		–5.8		–1.4		–1.3		–5.5		–2.2

CRSD, circadian rhythm sleep disorder; PSQI, Pittsburgh Sleep Quality Index; CI, confidence interval.

**Table 4.** Change scores and 95% confidence intervals for psychotic experiences and wellbeing measures

	Change scores													
	GPTS		SPEQ		DASS-D		DASS-A		DASS-S		WEMWBS		WSAS	
Comparison	Change	CI	Change	CI	Change	CI	Change	CI	Change	CI	Change	CI	Change	CI
Baseline *vs*	–13.4	–30.8,	–1.9	–5.6,	–2.9	–5.1,	–1.0	–3.7,	–4.0	–6.8,	5.7	1.4,	–5.5	–10.0,
post-therapy		4.1		1.7		–0.7		1.7		–1.2		10.0		–1.0
Baseline *vs*	–17.7	–29.9,	–4.5	–6.6,	–4.2	–6.0,	–0.9	–3.1,	–4.0	–6.2,	6.7	1.8,	–7.5	–13.7,
follow-up		–5.5		–2.3		–2.4		1.3		–1.8		11.6		–1.4

GPTS, Green Paranoid Thoughts Scale; SPEQ, Specific Psychotic Experiences Questionnaire; DASS, Depression Anxiety Stress Scale; WEMWBS, Warwick–Edinburgh Mental Well-Being Scale; WSAS, Work and Social Adjustment Scale; CI, confidence interval.

## Discussion

To our knowledge, this is the first study evaluating a CBT intervention for sleep problems in young people at ultra-high risk of psychosis. The aims were to establish the feasibility and acceptability of a brief, targeted intervention to improve sleep. Recruitment rates, treatment uptake and data completion were all high, which demonstrates the feasibility and acceptability of the intervention. In addition, the clinical outcomes indicate a potentially valuable intervention for this patient group: there were large effect size improvements in sleep and small to large effect size improvements in psychological wellbeing and psychotic experiences. Indeed at post-treatment the majority of participants were no longer in the clinical range for insomnia. These findings fit with the previous research literature: that sleep problems are common, that patients want treatment, and treatments can be successfully adapted across the spectrum of psychotic experiences. The key caveat is that this was an uncontrolled feasibility study.

### Limitations

There are clear limitations to this study. This was an uncontrolled feasibility study, therefore gains made post-treatment cannot be attributed with any certainty to the intervention. Whilst all follow-up assessments were rated by independent research assistants, they were not blind to whether sleep treatment had been provided, which is a cause of potential bias. The one-month follow-up showed that gains during the period of therapy were largely maintained and that no participants transitioned to psychosis; a further follow-up is needed to test longer term outcomes. For the next phase of research, an assessor-blind randomised controlled trial with a longer follow-up would establish the efficacy of the treatment. Completion of the sleep diary data was much lower than for the other measures. Utilizing technology, for example smartphone applications with automated reminders, may provide a solution to achieve daily sleep monitoring.

### Potential implications

Sleep problems represent an important treatment target for this age group, given the ongoing neurocognitive development and changes in sleep architecture that occur during this period. An intervention that is adapted specifically to address these changes and their effect on sleep problems may therefore be in high demand. Sleep disturbance also carries less stigma than other mental health difficulties. Given the ethical dilemmas associated with targeted prevention and terms such as ultra-high risk of psychosis, interventions that address sleep disturbance may be particularly beneficial. In this study, the intervention was popular, almost all of the participants completed the intervention, and all prospective participants deemed eligible for the study consented to take part. The intervention also has the advantage of being deliverable in a manualized format. This means it has the potential to be delivered by other mental health practitioners, allowing it to be more widely adopted by services.

Improving services for young people is at the forefront of mental health service delivery policy. Recent guidelines in the UK stipulate the provision of services for young people at ultra-high risk of psychosis (NICE, 2013; NHS England, 2015). As services seek to define what is beneficial and achievable for this group (Whale et al., 2017), a brief, targeted intervention that may have wider benefits could be of value. These wider benefits may include social and educational function and potentially preventing exacerbation of, or development of more severe, psychotic-like experiences.

### Clinical learning

Key adaptations for this age group to our treatment for sleep in psychosis included: addressing the social-environmental context and harnessing technology. The sleep environment was influenced by the developmental life stage of participants: most participants were either living with family or in student accommodation. This presented opportunities and challenges. Having a supportive network can help with the implementation of techniques and provide an external structure until new routines are embedded; however, participants were also seeking increased independence and thus the extent of involvement of others varied widely. The social lives and engagements of students are often at odds with consistent routines. We tried to address this by agreeing a period of experimentation in which the participant would maintain daytime activity but reduce the variability in their late-night activities. This was framed as an opportunity to re-set their sleep pattern, after which they would be more likely to ‘get away with’ the occasional late night. This was well-received by participants. Another challenge for students was that their entire living space (eating, sleeping, working and relaxing) might be confined to one room. In such cases, a hierarchy of sleep-promoting behaviour was developed (i.e. being off the bed when awake is better than sitting on the bed, which is better than lying on the bed). This allowed the student to follow the principles of the treatment as closely as possible. Finally, technology can be a distraction that delays sleep onset, but can also be a tool for establishing waking routines and monitoring sleep. Harnessing activity trackers and apps in psychological interventions is an area in which rapid progress is expected.

In summary, this study employed a brief intervention targeted at an identified causal mechanism in the development of psychotic experiences: sleep disturbance. The findings fit within a series of intervention studies that demonstrate the benefit of targeting sleep directly and the secondary benefits to psychological wellbeing and other mental health outcomes (Freeman et al., 2015, 2017). This study indicates that treating sleep problems in young people at ultra-high risk of psychosis is feasible, acceptable, and may be associated with benefits to sleep and psychological wellbeing.
